# Synchronous Measurements of Extracellular Action Potentials and Neurochemical Activity with Carbon Fiber Electrodes in Nonhuman Primates

**DOI:** 10.1523/ENEURO.0001-24.2024

**Published:** 2024-07-04

**Authors:** Usamma Amjad, Jiwon Choi, Daniel J. Gibson, Raymond Murray, Ann M. Graybiel, Helen N. Schwerdt

**Affiliations:** ^1^Department of Bioengineering, University of Pittsburgh, Pittsburgh, Pennsylvania 15213; ^2^Aligning Science Across Parkinson’s (ASAP) Collaborative Research Network, Chevy Chase, Maryland 20815; ^3^Department of Brain and Cognitive Sciences, McGovern Institute for Brain Research, Massachusetts Institute of Technology, Cambridge, Massachusetts 02139

**Keywords:** dopamine, electrophysiology, fast-scan cyclic voltammetry, multimodal electrochemical and electrical recording, neurotransmitters, striatum

## Abstract

Measuring the dynamic relationship between neuromodulators, such as dopamine, and neuronal action potentials is imperative to understand how these fundamental modes of neural signaling interact to mediate behavior. We developed methods to measure concurrently dopamine and extracellular action potentials (i.e., spikes) in monkeys. Standard fast-scan cyclic voltammetric (FSCV) electrochemical (EChem) and electrophysiological (EPhys) recording systems are combined and used to collect spike and dopamine signals, respectively, from an array of carbon fiber (CF) sensors implanted in the monkey striatum. FSCV requires the application of small voltages at the implanted sensors to measure redox currents generated from target molecules, such as dopamine. These applied voltages create artifacts at neighboring EPhys measurement sensors which may lead to misclassification of these signals as physiological spikes. Therefore, simple automated temporal interpolation algorithms were designed to remove these artifacts and enable accurate spike extraction. We validated these methods using simulated artifacts and demonstrated an average spike recovery rate of 84.5%. We identified and discriminated cell type-specific units in the monkey striatum that were shown to correlate to specific behavioral task parameters related to reward size and eye movement direction. Synchronously recorded spike and dopamine signals displayed contrasting relations to the task variables, suggesting a complex relationship between these two modes of neural signaling. Future application of our methods will help advance our understanding of the interactions between neuromodulator signaling and neuronal activity, to elucidate more detailed mechanisms of neural circuitry and plasticity mediating behaviors in health and in disease.

## Significance Statement

We present a simple method for recording synchronous molecular and neuronal spike signals. Conventional electrophysiological and electrochemical instruments are combined without the need for additional hardware. A custom-designed algorithm was made and validated for extracting neuronal action potential signals with high fidelity. We were able to compute cell type-specific spike activity along with molecular dopamine signals related to reward and movement behaviors from measurements made in the monkey striatum. Such combined measurements of neurochemical and extracellular action potentials may help pave the way to elucidating mechanisms of plasticity and how neuromodulators and neurons are orchestrated to mediate behavior.

## Introduction

Most neurons communicate with each other via both chemical and electrical forms of signaling. The relative timing between dopamine neurotransmitter release and action potentials of target neurons has been shown to regulate synaptic plasticity (Reynolds et al., 2001; Yagishita et al., 2014; Brzosko et al., 2019; Shindou et al., 2019). Thus, measuring how chemical and electrical neural signals are coordinated during online behavior is central to studying brain function and the multimodal mechanisms that shape how neural circuits are programmed to regulate behavior.

Nevertheless, most of our understanding of brain function comes from measurements of either chemical or electrical signals, but not both. Such single mode measurements preclude the ability to look at how molecules, such as dopamine neurotransmitters, modulate nearby neuronal spike activity and vice versa—how neuronal activity influences extracellular molecular signaling. Furthermore, there is a critical gap in our knowledge of how such signals are coordinated to mediate behavior. Thus, dual electrical and chemical measurements are imperative to dissect these interactions, especially in awake behaving animals.

Concurrent measurements of electrical and chemical neural signals may be achieved by combining standard electrophysiology (EPhys) with molecular recording techniques. Molecular recording may involve electrochemical or optical sensors that provide the needed millisecond temporal resolution and microscale dimensions to capture the fast dynamics of neurochemical release and clearance (Rice et al., 2011). Optical methods have advanced immensely in use over the past few years, demonstrating remarkable specificity for a variety of neurotransmitters and neuromodulators such as dopamine, serotonin, acetylcholine, and many other compounds (Patriarchi et al., 2018; Sun et al., 2018; Jing et al., 2020; Unger et al., 2020). These methods require genetic modification of neurons to express synthetic fluorescent receptors (e.g., dLight and GRABDA) and/or introduction of exogenous compounds (e.g., fluorescent false neurotransmitters; Gubernator et al., 2009; Pereira et al., 2016) or cells (e.g., CNiFER; Q-T. Nguyen et al., 2010). Nevertheless, application in nonhuman primates has been limited, and such genetic modification is ethically prohibited in humans.

This work uses electrochemical (EChem) methods, specifically, fast-scan cyclic voltammetry (FSCV). FSCV has been established over several decades to record current generated through reduction and oxidation (i.e., redox) of dopamine and other chemical compounds (Kawagoe et al., 1993; Clark et al., 2010). A voltage scan is applied to the implanted electrode to generate this redox current that is proportional to the concentration of the analyte. Carbon fiber (CF) electrodes (CFEs) have been the mainstay for such sensors given their high electron transfer, biocompatibility, and adsorptive properties (McCreery, 2008). FSCV has demonstrated robust performance for chronic measurements in rodents (Clark et al., 2010; Schwerdt et al., 2018) and monkeys (Schwerdt et al., 2017, 2020) and has even been applied in humans intraoperatively (Kishida et al., 2016). Thus, the realization of its potential for clinical translation is imminent.

**Figure 1.**
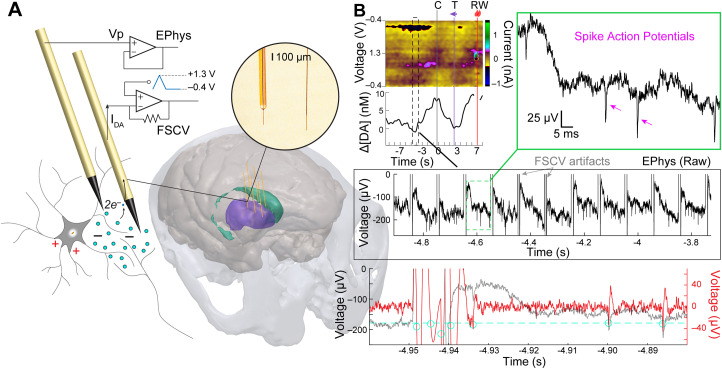
***A***, ECP recording setup for synchronous recording of dopamine and neuronal action potentials as recorded from separate CF electrodes implanted in the monkey striatum (colored purple and green) and connected to electrochemical (FSCV) and EPhys recording systems, respectively. ***B***, Example recordings from ECP system in a task-performing monkey. FSCV-recorded dopamine signals are plotted as a function of time as displayed on a color plot where current changes (color scale) are clearly observed at the redox potentials for dopamine (∼−0.2 and 0.6 V) and its PCA-extracted dopamine concentration change ([ΔDA]) below it. Task events for the display of the initial central cue (C), peripheral reward-predictive target (T), and reward delivery (RW) are displayed as vertical lines. Below this, the concurrent EPhys recording is shown for a small time window (black dashed rectangle) during the dopamine trace, showing the interfering FSCV scan artifacts. A close-up of the EPhys recording between two scan artifacts (green dashed rectangle) is shown to visualize clear spike action potentials (arrowheads on top right inset). The bottom panel shows the signal after high-pass filtering at 250 Hz using forward-only filters as would be applied for standard spike detection algorithms. This period includes a close-up of the scan artifact demonstrating its triggering of multiple threshold (dashed line)-crossings (circles). Three physiological units are also detected but the first of these is largely distorted by the forward-filtering of the artifact.

Here, we combine EChem and EPhys to measure molecular and electrical neuronal activity, respectively, and this will be referred to hereon as ECP (i.e., electrochemical and electrophysiological) recording for simplicity (Fig. 1). ECP measurements are not straightforward as FSCV operation requires application of scan voltages at implanted sensors, and these FSCV scans are directly picked up by neighboring EPhys recording sensors in the form of voltage artifacts (Fig. 1*B*). Removing these artifacts from the analysis is imperative to compute accurate local field potential (LFP) and spike information in the EPhys data. Scan artifacts can be erroneously labeled as spikes using standard thresholding and clustering-based spike sorting methods (Schmitzer-Torbert and Redish, 2004; Schmitzer-Torbert et al., 2005). Consequent analytical measures, like spike firing rates, are then susceptible to error and misrepresentation of the actual neural environment.

In this work, we developed a temporal interpolation algorithm to extract neuronal spike activity from ECP recordings without the need for additional hardware (Parent et al., 2017). We leveraged the periodicity of the FSCV scans to detect the timing of these signals and interpolate their generated artifacts in the EPhys recording. The algorithm was extended for use to remove line noise at 60 Hz and its harmonics. We characterized the recovery rate of our spike extraction techniques by simulating FSCV scan artifacts onto isolated EPhys recordings, demonstrating the ability to extract a large percentage (84.5%) of the original spikes. We were able to capture behaviorally relevant computations of synchronous dopamine and spike activity from functionally diverse neurons as measured from the monkey striatum. The techniques described here introduce a new way to measure and analyze the interactive relationship between neuromodulator signaling and surrounding neuronal activity and how these coordinate activities influence and/or are shaped by behavior.

## Materials and Methods

### Animal procedures

Measurements and experimental methods used in this study were obtained from one female rhesus monkey (∼8.5 years old and weighing ∼10 kg at time of recordings). All experimental procedures were approved by the Committee on Animal Care of the Massachusetts Institute of Technology and are described in a previous report (Schwerdt et al., 2020).

### Behavioral task

The monkey performed a visually guided reward-biased task, as described previously (Schwerdt et al., 2020), during all data collection reported in this work. Briefly, each trial consisted of an initial central cue (C) that the animal had to saccade to and fixate on for 1.2–3 s. A peripheral target (T) cue appeared on the left or right of the screen after the C extinguished. The animal had to saccade and fixate on the T cue for 4 s to receive a small or large reward (RW), which consisted of 0.1–0.3 or 1.5–2.8 ml of liquid food, respectively. The association between target direction (left or right) and reward size (small or large) was switched every 20–30 trials. These methods are also described at protocols.io (https://doi.org/10.17504/protocols.io.kqdg325eev25/v1).

### ECP recording setup

Both EPhys and EChem-FSCV measurements were made from eight or nine chronically implanted CF sensors, fabricated following previously published methods (Schwerdt et al., 2017), implanted in the monkey striatum (Fig. 1). The setup is described in a previous publication (Schwerdt et al., 2020) and a brief summary is provided here. CF electrodes consisted of either conventional silica tube-threaded electrodes (silica-CF) or parylene-encapsulated electrodes (py-CF). CF sensors were mounted onto microdrives atop a custom-designed chamber (Gray Matter Research) that was affixed to the monkey's skull. The sensors were inserted into the brain through guide tubes (Connecticut Hypodermics, 27G XTW) and lowered to the striatum (i.e., caudate nucleus, CN, and putamen). Sensors were individually connected to either standard electrophysiological (EPhys) recording system (NeuraLynx, HS-32) or an FSCV system (obtained from S.B. Ng-Evans at University of Washington) as selected on a day-by-day basis. EPhys signals were referenced to multiple tied stainless-steel wires inserted in the tissue above the dura mater (A-M Systems, 790700). Ag/AgCl electrodes were implanted in the epidural tissue and/or in a white matter brain region to serve as the FSCV reference.

Dopamine concentration changes were recorded from implanted sensors using a four-channel FSCV system (from S.B. Ng-Evans at University of Washington). This consisted of a transimpedance amplifier headstage to convert and amplify electrochemical current to voltage and a computer to control the applied voltage scans and record and store current. This system generated a triangular voltage ramping from −0.4 to 1.3 V and back to −0.4 V, with a scan rate of 400 V/s. This was applied at a sampling frequency of 10 Hz to the connected electrodes. A holding potential of −0.4 V was maintained at the electrode between scans. Background-subtracted color plots were generated by plotting the relative current change as color, applied scan voltage on the *y*-axis (i.e., −0.4 to 1.3 to −0.4 V), and time (i.e., each scan at 100 ms intervals) on the *x*-axis. Chemical specificity is determined by the redox voltages, and these voltages are also dependent on the sensor material and scan parameters. Parameters optimal for selective and sensitive dopamine detection have been established over several decades (Keithley and Wightman, 2011; Rodeberg et al., 2017).

EPhys recording was performed using a standard electrophysiology system (NeuraLynx, HS-32). The recording settings were configured as follows: input range of ±1 mV, sampling rate of 30 kHz, and bandpass filter with a passband at 0.1–7,500 Hz. This system received the timestamps for behavioral task events as generated from the VCortex (National Institute of Mental Health) behavioral system. A digital messaging system (NeuraLynx, NetCom Router) allowed transmitting of trial-start events from the EPhys system to the FSCV system to provide shared timestamps between the FSCV and EPhys systems.

Here, we recorded FSCV and EPhys on separate sensors for what we refer to as a decoupled configuration. Recordings may also be made simultaneously from the same sensor (Cheer et al., 2005; Takmakov et al., 2011), but this usually requires actively switching between the recording modalities so that the applied voltage from FSCV does not overly saturate and/or damage the input amplifiers on the EPhys system. This temporal multiplexing is possible because FSCV parameters used for dopamine detection only apply the scan to the sensor during an 8.5 ms period of the 100 ms sampling interval; the sensor can be disconnected from the FSCV input during the remaining 91.5 ms period to allow EPhys recording. Typically, the interval outside of the scan is used to hold a negative potential (e.g., −0.4 V) to attract positively charged molecules such as dopamine to the CF in between scans (Bath et al., 2000). Applying this hold potential in a single-sensor configuration would effectively short the EPhys input throughout the entire sampling interval, preventing any EPhys measurement.

A decoupled ECP configuration, as applied here, permits applying a holding potential and, in addition, may remove the need for supplementary circuits to switch between EPhys and FSCV operations since applied scan voltages attenuate with distance to other implanted sensors. Nevertheless, the FSCV voltage scanning artifacts are still recorded by neighboring EPhys sensors due to the conductive nature of the brain tissue. The spacing between electrodes varied between 1 and 6 mm, as described in our previous publication, where a map of their estimated anatomical positions within the striatum may also be found (Schwerdt et al., 2020). These methods are also described at protocols.io (https://doi.org/10.17504/protocols.io.yxmvme785g3p/v1).

### Dopamine concentration estimation

Signals recorded from the FSCV system were processed in MATLAB (2023b; RRID:SCR_001622; http://www.mathworks.com/products/matlab/) to extract targeted dopamine concentration changes ([ΔDA]) using principal component analysis (PCA), as previously described (Schwerdt et al., 2017). Briefly, each FSCV scan produces a cyclic voltammogram (CV; i.e., current vs voltage plot), which is background subtracted to remove the larger current contributions associated mainly with nonfaradaic processes and to distinguish the smaller current changes related to chemical redox. The background subtraction usually occurs at an arbitrary reference time (i.e., alignment event such as the T cue) to provide a uniform reference for all task-modulated signals on each trial. These background-subtracted currents are projected onto the principal components computed from standards of dopamine, pH, and movement artifact as previously established (Schwerdt et al., 2017). CVs that produced an excessive variance (*Q*) above a tolerance level (*Q_α_*) could not be accounted as a physiological signal and were nulled automatically (assigned NaN values in MATLAB). Signals were also nulled where CVs were correlated to movement artifact standards (*r* > 0.8). These strict procedures ensure that at least 90% of the signals are identified as dopamine, with a false-negative rate of <30% and 0% false positives.

### Temporal interpolation methods to extract spike data

**Figure 2.**
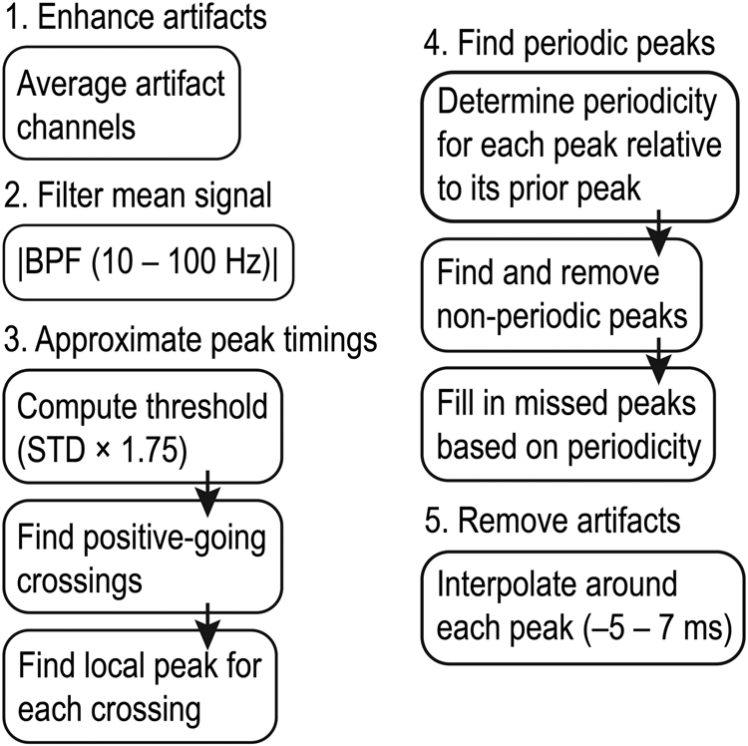
Flow chart of temporal interpolation algorithm used to extract spike activity from ECP recordings. Details may be found in the text.

We developed a simple automated algorithm to reliably extract extracellular action potentials by performing linear interpolation over the FSCV artifacts embedded in the EPhys waveforms in the time domain. These methods were also extended to remove other sources of periodic noise, such as line noise at 60 Hz and its harmonic frequencies. This algorithm is applied on each session independently to detect the artifact peak timings specific to an individual recording. Artifacts are identified by finding signals that exceed a threshold. The artifact shape and amplitude depends on a number of factors, including the distance between the FSCV recording electrodes and the EPhys electrodes, as well as the characteristics of the EPhys electrodes (e.g., impedance and other nonlinear electrode–tissue interface properties; Nag et al., 2015). The threshold crossings are attributed to FSCV scans by relying on their known periodicity (e.g., 10 Hz for FSCV or 60 Hz and its harmonics in the case of line noise), to prevent false identification of other threshold crossings. These detection methods are preceded by high-pass filtering the signal first to isolate the sharp peaks generated by the FSCV scans and to prevent threshold crossings caused by low-frequency LFP signals or other slow voltage shifts. Also, signals from multiple recording channels are averaged together to suppress signals unrelated to common noise or artifact coupling and as a result, enhance the targeted artifacts for ease of detection. The original signals are then interpolated across the time windows in which artifacts are detected. This interpolation is necessary to prevent falsely attributing the artifacts to physiological spikes, in subsequent spike sorting steps (described in the subsequent section) as is seen to occur in the noninterpolated signal displayed in Figure 2*B*. The specific details of these algorithms are described below.

**Figure 3.**
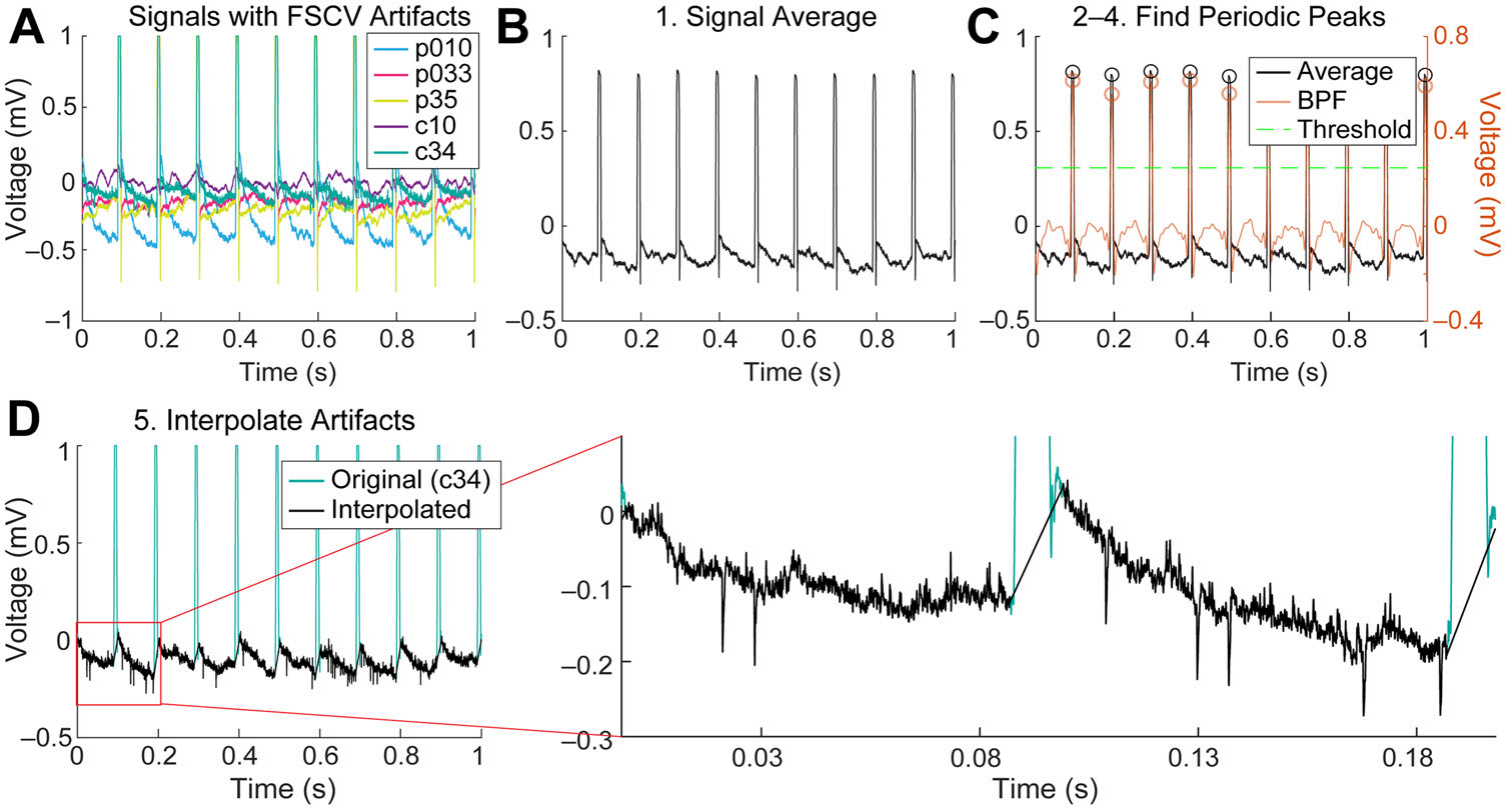
Temporal interpolation process applied to an example ECP recording. ***A***, Signals from five electrodes containing FSCV scan artifacts, electrode sites are labeled in the legend (c denotes CN site and p denotes putamen site). ***B***, All these signals are then averaged (step 1 in interpolation algorithm). ***C***, The averaged signal is bandpass filtered (BPF) and a threshold is computed (1.75 × STD) to find the positive-crossings and local peaks for each of these (circles). Only periodic peaks are retained (or added if they did not cross the threshold initially). ***D***, Linear interpolation is performed around each of these identified peaks using a window of −3 to 7 ms for each electrode channel. An example is shown for site c34 in the bottom left plot, as well as a close-up on the bottom right, to visualize recorded unit spike activity.

Our algorithms were implemented in MATLAB (MathWorks, 2023b). A flowchart of the algorithm is illustrated in Figure 2 and details are as follows. (1) The first step in the algorithm is to get a first approximation of the timings of the artifacts. Signals from multiple (up to 5) EPhys electrodes are averaged to enhance the artifact peaks that are coherent across channels and to reduce background spike and LFP fluctuations that may inadvertently trigger threshold crossings used in subsequent steps (Fig. 3*A*,*B*). We also found that using signals from a single EPhys electrode was sufficient to produce the same performance in emulated data (i.e., the spike recovery rate metric described in Results, Validation of spike extraction methods). (2) The averaged signal is bandpass filtered (BPF) at 10–100 Hz, and then its absolute value is taken. This procedure further enhances the artifact peaks over surrounding signals. (3) The artifact peak timings are identified by first finding positive-going crossings over a threshold, a multiple (i.e., 1.75) of the standard deviation of the previously calculated signal. The local peak directly following each positive crossing is identified and stored. (4) Only peaks demonstrating periodicity according to the expected FSCV scan frequency (10 Hz) are retained to prevent unwanted removal of physiological signals. This is done by checking the periodicity of each peak relative to its prior peak. Any nonperiodic peak is removed from the stored variable containing a list of threshold crossing peaks. Furthermore, missing peaks are added based on the periodicity of the peaks captured before or after the window in which peaks were not detected. This ensures that artifacts that exist below the initial detection threshold are captured and resolved to minimize erroneous physiological data. (5) Finally, linear interpolation is performed on the signal around each identified peak with a window of −5 to 7 ms. This window was empirically determined through incremental (0.5 ms) increases beyond the defined 8.5 ms scan window applied in FSCV. The wider window accounts for recovery time of the amplifier as well as low-pass filtering and resulting broadening of the signal through the tissue. This interpolation removes the artifact, preventing it from being detected falsely as a spike during subsequent high-pass filtering and thresholding steps used for spike sorting.

This algorithm was further extended to remove artifacts associated with line noise at 60 Hz and its harmonic frequencies (e.g., 120 Hz; Extended Data Fig. 3-1). Such line noise interference was frequently present in our experimental setup, most likely due to the shared ground between EPhys and EChem systems. Removing this interference only required a simple modification to the previous algorithm, where the filtering process in Step 2 was replaced by a high-pass filter (300 Hz cutoff) as this accounted for the higher-frequency content of the noise and that the threshold computation was performed by taking a multiple (i.e., 8) of the average of the filtered signal rather than its standard deviation. Also, the interpolation window in Step 5 was reduced to −0.166 to 0.166 ms as these signals were of much shorter duration than the FSCV scans. These artifacts were found to be smaller and frequently within the range of actual physiological spikes (∼100 µV) in some electrodes. Averaging multiple electrodes in the first step of our algorithm was useful to enhance these periodic signals above the variable background signaling to ensure their detection and interpolation.

10.1523/ENEURO.0001-24.2024.f3-1 Figure 3-1 Same as Fig. 3 but demonstrating an example with 60  Hz + harmonics noise and the additional steps to remove these signals. (D) After high pass filtering, the noise was enhanced. (E) Unit spike activity is more clearly discernible after interpolating both 60  Hz harmonics noise and the FSCV artifacts. Download Figure 3-1, TIF file.

These interpolation algorithms do not require a clock input to provide timings of the FSCV scans and instead relies on the periodicity of the signal and its appearance on multiple EPhys recording electrodes. The periodicity of the signal is a key input into the algorithm as simple thresholding methods used to remove large glitches or artifacts may inadvertently remove physiological signals. Template methods (Hashimoto et al., 2002; Wichmann and Devergnas, 2011; Nag et al., 2015; O’Shea and Shenoy, 2018; Banaie Boroujeni et al., 2020) frequently used for removing artifacts caused by electrical stimulation are not able to account for the range of variability in the shape of the FSCV artifact, which depends on the distance between the electrodes and the electrode properties. These algorithms may be found at GitHub as made available through zenodo (https://doi.org/10.5281/zenodo.10955584).

### Spike sorting

Spike sorting was done on the interpolated EPhys signals (as described in the preceding section) as well as noninterpolated signals collected during ECP recording from multiple striatal electrodes in task-performing monkeys. The spikes extracted from the EPhys signals displayed modulation to several behavioral task variables (as described in Results, Measurements of cell selective spike activity) and could then be compared side-by-side with synchronously recorded dopamine measurements (as described in Results, Behaviorally relevant measurements of dopamine and spike coactivity). Spike sorting was performed manually in commercial software (Plexon, Offline Sorter 4.6.2; RRID:SCR_000012; http://www.plexon.com/products/offline-sorter) following standard protocols (Feingold et al., 2012). Raw EPhys data were first high-pass filtered using a Butterworth filter with a cutoff frequency of 250 Hz (four-pole, forward only). A negative threshold was set to identify negative-going crossings of the action potential waveforms. Waveforms were extracted with a length of 1.6 ms (48 samples) using a prethreshold period of 0.267 ms (8 samples). These waveforms were then visualized in terms of their energy, nonlinear energy, and their projections onto principal component (PC) space (e.g., PC1 vs PC2). Waveforms were first invalidated based on the prominence of energy and nonlinear energy features, which enhances visualization of artifact-like signals such as large transient glitches created by electrostatic discharge as well as from the reward delivery peristaltic pump. Waveforms were then manually invalidated in the PC space if they looked like the artifacts mentioned previously and did not resemble physiological spike signals (e.g., high-frequency changes in voltage). PCs were recalculated after invalidating waveforms to distinguish potential clusters based on the variances calculated for valid waveforms. Clusters were manually drawn on the PC space when a distinct boundary was observed between the projected points. These clustered waveforms were then exported for plotting average waveforms and histograms in MATLAB. Interspike interval (ISI) histograms were generated using 1 or 2 ms bin widths to visualize the relative distribution of spike firing intervals, which is used as a parameter to distinguish different cell types (Aosaki et al., 1994).

## Results

### Validation of spike extraction methods

**Figure 4.**
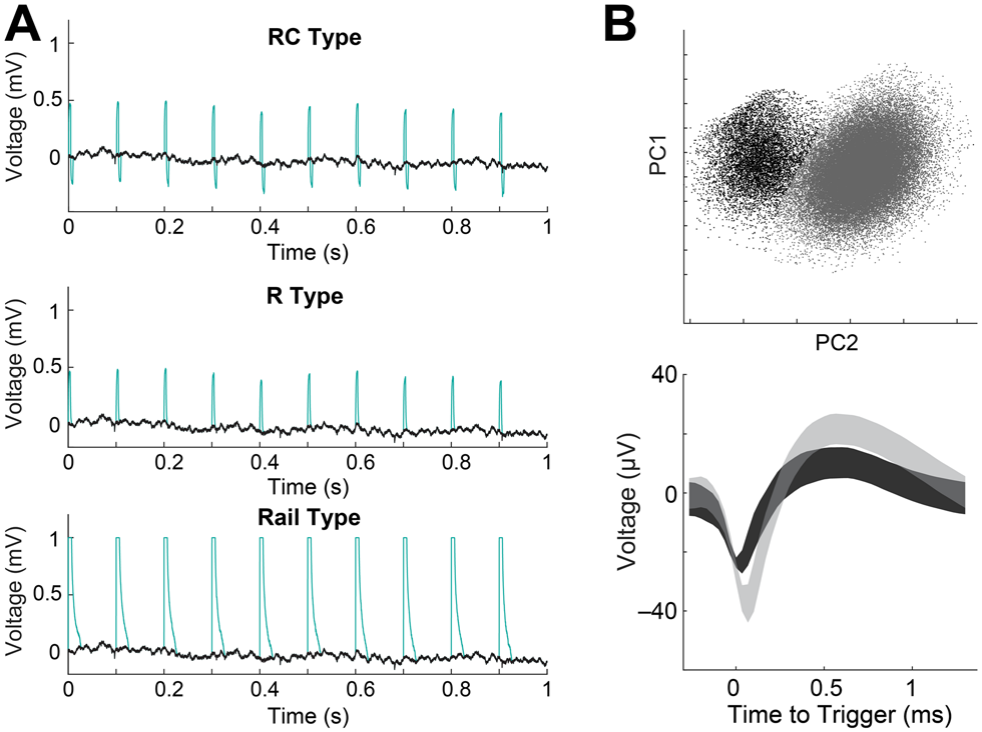
***A***, Validation setup showing three different simulated FSCV artifact types (RC, R, Rail; green trace) added onto EPhys-only recording (black trace; session 65B and site p32). ***B***, Top, Spike waveforms projected onto PC space (top) with colored clusters. Bottom, Average spike waveforms for drawn clusters from the PC space (top). Shading represents ± SD.

Our algorithm was validated by adding synthetic FSCV artifacts onto EPhys recordings made without FSCV (i.e., EPhys-only; Fig. 4). FSCV artifacts were emulated following methods previously established for validating LFP extraction algorithms in similar ECP measurement configurations (Schwerdt et al., 2020). Three different types of artifacts were simulated: resistive (R), resistive-capacitive (RC), and rail (i.e., saturating). These differences reflect characteristics of the artifact duration and amplitude observed in EPhys recordings made during concurrent FSCV and arise due to differences in distance between electrodes, as well as the electrode properties. Spike sorting was done on the EPhys-only recording, before adding artifacts, and then again after adding artifacts and implementing our interpolation algorithms. Spike recovery rate was defined as the percent of physiological spikes retained after interpolating the artifacts.

Simulation of each of the three types of artifacts was implemented individually on five recorded channels from three different sessions, and the results are shown in Table 1. We calculated the spike recovery rate for each type of artifact being interpolated with our algorithm. We found that the spike recovery rate was 84.5% as averaged for all artifact types. This is equivalent to a loss of 15.5%, which was a reasonable result given that the 8.5 ms FSCV scans make up 8.5% of the recording, and our interpolation occurs over a wider 12 ms and therefore 12% of the recording. The average rates for interpolating each type of artifact were 88, 85.8, and 79.6% for the R, RC, and rail-type artifacts. Spike recovery rates were the worst for interpolating rail-type artifacts as expected given their broader width that encompasses the amplifier recovery time after its input saturates. This is important to consider when designing higher-density configurations of CF sensor arrays as minimizing the distance between sensors will increase the artifact amplitudes. These performance metrics were virtually unchanged when only a single EPhys electrode was used for the artifact averaging process in the first step of our algorithm (described in Materials and Methods, Temporal interpolation methods to extract spike data). However, it is possible that these artifacts may have significantly reduced amplitudes in sensors further from FSCV recording electrodes, which we did not test in our current configuration. On the other hand, the artifacts related to line noise were smaller, within the range of actual physiological spikes (∼100 µV), and more variable in amplitude (Extended Data Fig. 3-1). Therefore, these artifacts were frequently missed by our algorithm when only a single EPhys electrode was used. Maximizing the number of electrodes used for averaging, to reduce the amplitude of signals variable across channels and enhance the artifacts that are concurrent across channels, helped ensure that these artifacts were captured and interpolated by the algorithm. Code and data used for simulating artifacts and validation are available at GitHub and zenodo (https://doi.org/10.5281/zenodo.10955525 and https://doi.org/10.5281/zenodo.10396372).

**Table 1.** Validation results showing percent recovery of spikes extracted after interpolating simulated artifacts relative to spikes extracted from clean EPhys recording for the different types of artifacts (R, RC, and Rail)

Session	Channel	R recovery	RC recovery	Rail recovery
65B	p23	87.13	86.39	79.55
	p32	87.23	86.9	83.51
109B	p35	88.66	86.07	76.07
	c34	91.78	84.48	81.02
161b	p36	85.35	85.06	77.94

### Measurements of cell selective spike activity

**Figure 5.**
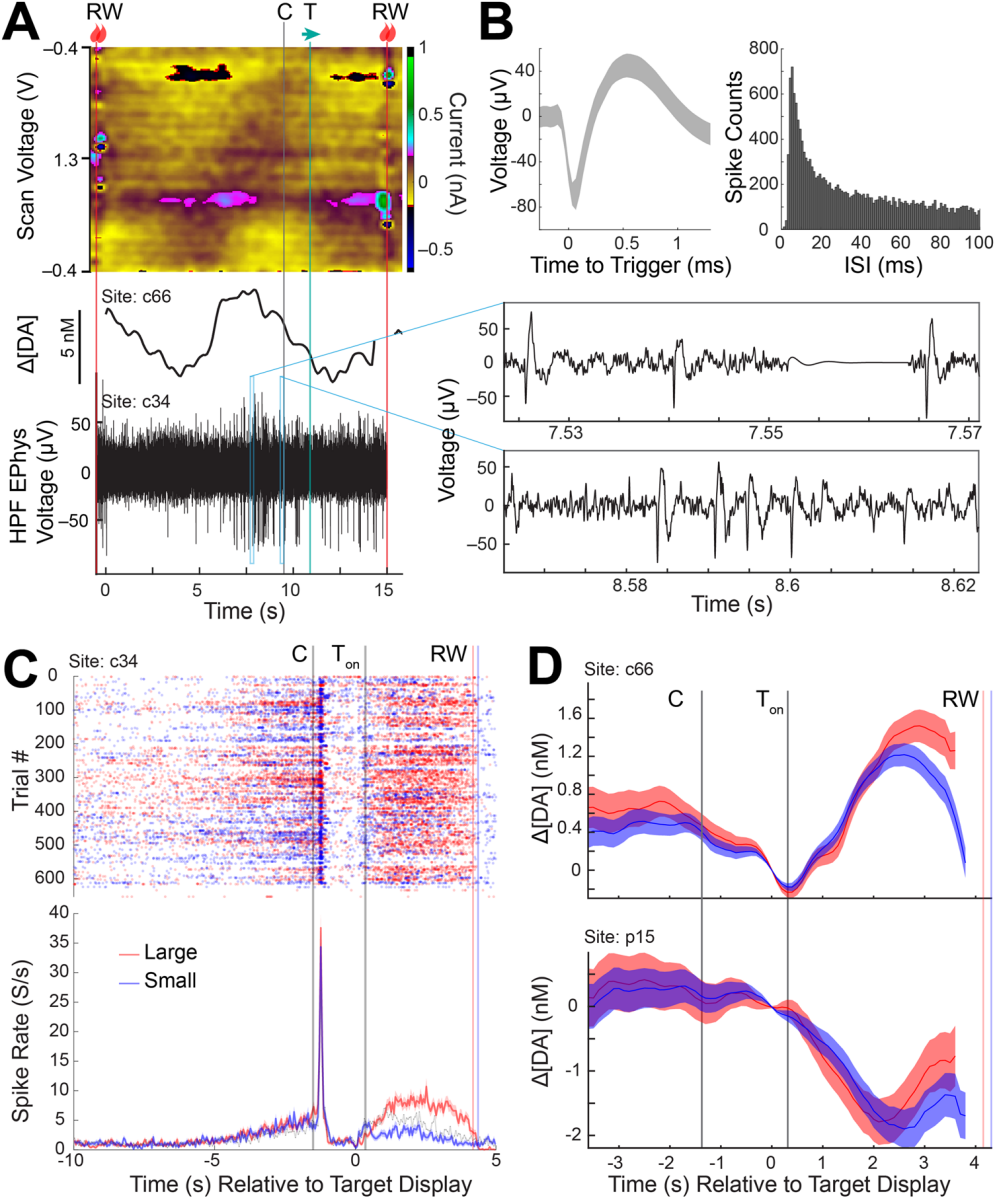
Analysis of task modulated-signaling from concurrent recordings of dopamine and spike activity from three sites in the CN and putamen measured from a single session (session 127). ***A***, Top, FSCV color plot; middle, PCA-computed dopamine concentration change ([ΔDA]); and bottom, concurrent measurements of electrical neural activity high-pass filtered (HPF) to visualize spike action potentials. Two windows (blue rectangles) are magnified to show the individual spike action potential waveforms (right). Task events are labeled following notation in Figure 1. ***B***, Left, Average waveform of unit detected (shading represents ± SD). Right, ISI histogram of the detected unit. ***C***, Top, Trial-by-trial raster plot of spike activity (dot) measured in the CN (c34) as aligned to the peripheral target display event (0 s). T_on_ represents the average time from the peripheral target display event at which the monkey begins fixation on the peripheral target. Bottom, Average spike rate for large and small reward trial conditions (shading represents ± SE). Large and small reward trials are denoted by red and blue colors, respectively. ***D***, Dopamine concentration changes measured at neighboring sites in the CN (c66) and putamen (p15) as aligned to the same events as ***C*** for large and small reward conditions (shading represents ± SE). Color coding is the same as in ***C***.

**Figure 6.**
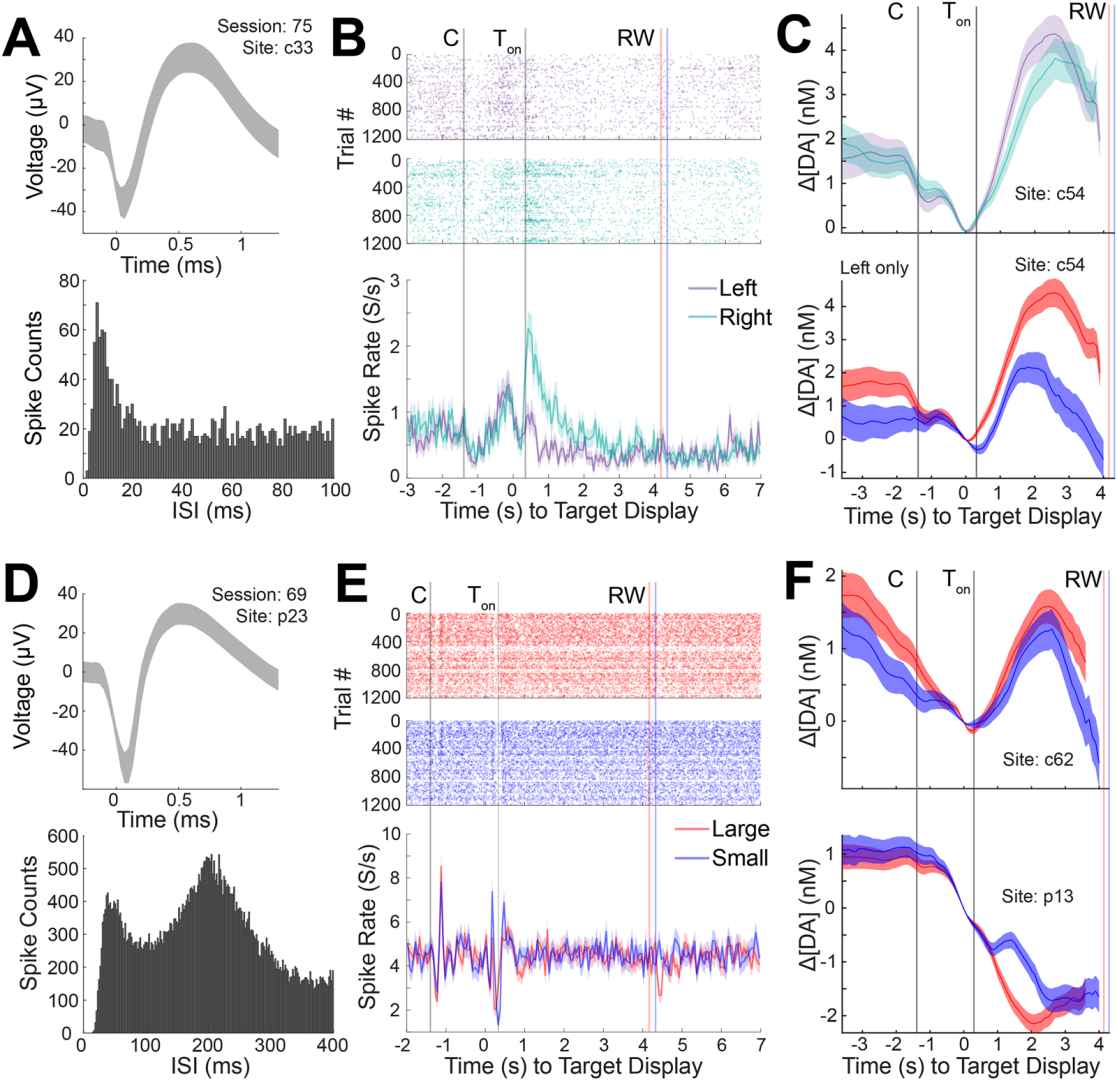
Dopamine and spike activity recorded concurrently from a single session show diverse responses to behavioral events related to reward size and spatial target direction. ***A***, Average waveform of a putative MSN (top) and its ISI histogram (bottom) as recorded in the CN (c33). ***B***, Raster plot of spike activity for the unit in ***A*** as plotted in Figure 5*C*, except for left (purple) and right (green) peripheral target conditions, demonstrating higher neural responses to gaze of the right peripheral targets than to left targets. ***C***, Dopamine concentration changes measured at a neighboring site in the CN (c54) displaying oppositive sensitivity to target direction (higher for left than for right target) in comparison with the unit in ***B*** (top; color coding is the same as in ***B***), and stronger modulation by reward size (bottom; color coding is the same as in Fig. 5*C*). Only left target conditions are plotted on the bottom panel as reward sensitivity was not observed in right target conditions. ***D***, Same as ***A*** except for another unit (putative TAN) recorded in a separate session (69) and site in the putamen (p23). ***E***, Same as ***B*** except for large (red) and small (blue) reward conditions. No distinction is observed in the cell firing for the reward size or target direction. ***F***, Dopamine concentration changes measured at neighboring sites in the CN (c62) and putamen (p13) where stronger modulation by reward size is observed in comparison with the unit response in ***E***. Color coding is the same as in ***E***.

Extracted spike activity was analyzed as measured from implanted CF sensors in the CN and putamen during concurrent FSCV in a task-performing monkey (Figs. 5, 6). Striatal units have been shown to display specific waveform shapes and firing characteristics depending on the cell type classification (Hikosaka et al., 1989; Apicella, 2002; Yamada et al., 2004; Thorn et al., 2010). Our units displayed distinct characteristics that largely resembled either putative medium spiny neurons (MSNs) or tonically active neurons (TANs), as classified in prior work. TANs display a longer after-hyperpolarization (i.e., broader shape; Fig. 6*E*) in comparison with MSNs (Fig. 5*B*; Hikosaka et al., 1989; Apicella, 2002). Furthermore, MSNs are known to fire scarcely (<1 Hz) and increase sharply (i.e., burst) in response to relevant behavioral events, which is noticed in the peak spike counts among the low interspike intervals (ISIs) in the plotted ISI histograms (Figs. 5*B*, 6*B*). On the other hand, TANs maintain spontaneous firing rates of 2–12 Hz and show transient pauses in response to a variety of stimuli or events (Hikosaka et al., 1989; Aosaki et al., 1994, 1995; Apicella, 2002). TANs may also display up to two peaks in their ISI histograms (Aosaki et al., 1994), which was also observed in our unit (Fig. 6*E*). These cell type distinctions may also be perceived in the spike rate histograms plotted in association with behavioral events (Figs. 5*C*; 6*A*,*D*), where the average spontaneous firing rates outside of trial bounds (i.e., before the central start cue and after the outcome) are higher for putative TANs (Fig. 6*D*) than for MSNs (Figs. 5*C*, 6*A*). The event-related discharges and pauses of putative MSNs and TANs, respectively, may be observed in these plots. These results collectively demonstrate our ability to resolve cell type-specific spike activity as measured during concurrent FSCV electrochemical recording.

### Behaviorally relevant measurements of dopamine and spike coactivity

We analyzed how synchronously recorded dopamine and spike activity were modulated by behavioral task events related to reward size, eye movement direction, and visual cues. Multimodal measurements were made in a monkey performing a task where eye movements were made to left or right targets to receive liquid-food rewards. The size of the reward (i.e., large or small) depended on the target location (i.e., left or right), and this was switched every block to counterbalance reward size and movement direction variables. More details of the task are described above in Materials and Methods, Behavioral task.

Spike activity of a putative MSN recorded in the CN (c34) was shown to increase significantly in response to the appearance of the initial central start cue. This neuron also showed significant modulation by the size (large or small) of the upcoming reward (Fig. 5*C*). Such reward size modulation is similar to that observed in prior experiments (Cromwell and Schultz, 2003; Kawagoe et al., 2004). These reward size activities were maintained for a sustained period throughout the period from the target cue onset to the reward delivery, possibly reflecting ongoing functions related to invigoration (Cromwell and Schultz, 2003). Furthermore, pre-cue anticipatory activity is observed in this neuron, also seen in previous work (Kawagoe et al., 2004) and has been linked to facilitating subsequent movements. We found that dopamine was also modulated by reward size anticipation in a neighboring site (c66) in the CN, but not in our putamen site (p15; Fig. 5*D*). Dopamine signals were not modulated by the initial central cue in either site.

In another CN site (c33) and session, a putative MSN's spike activity was found to be target direction sensitive, displaying higher firing rates during gazes to right (ipsilateral) targets in comparison with left (Fig. 6*A*). Unlike the previous unit, this neuron was not modulated by reward size. Such spatial selectivity has been observed of striatal units recorded in similar eye movement tasks (Kawagoe et al., 2004). Synchronously recorded dopamine signals, on the other hand, were modulated by both reward size and target direction (Fig. 6*C*). However, dopamine was slightly higher for contralateral targets.

A putative TAN recorded in the putamen (p23), also in a separate session, displayed transient decreases in spike firing in response to displayed visual cues (i.e., initial central cue and peripheral target) followed by a rebound increase, replicating event-related pause activity frequently observed in TANs (Fig. 6*D*; Hikosaka et al., 1989; Aosaki et al., 1994, 1995; Apicella, 2002). The neuron did not discriminate between reward size or movement direction. These patterns of observations are in line with prior work (Hikosaka et al., 1989; Apicella, 2002; Yamada et al., 2004; Thorn et al., 2010), which have largely attributed these signals to representing the motivational value or arousing aspects of external stimuli. Striatal dopamine in the putamen and CN was modulated by reward and target cue direction (Fig. 6*F*), similar to our previous examples and other reports (Schwerdt et al., 2020).

## Discussion

Methods for recording and analyzing extracellular action potentials as measured concomitantly with FSCV-based dopamine signals were developed and validated in this study. Minimal additional hardware was required beyond standard EPhys and FSCV instrumentation to develop our ECP system. Spike extraction was carried out off-line using simple custom-made temporal interpolation algorithms. These algorithms leveraged the periodicity of the FSCV voltage scans to remove the interfering FSCV voltage scan artifacts off the EPhys recordings. We validated the extraction technique using artificial artifacts to ensure that spikes were retained with high fidelity. ECP recording was performed on CF sensors implanted in the striatum of a monkey to uncover the coactive dopamine and spike signals underlying reward and movement behaviors. We further demonstrated the ability to distinguish different neuronal cell types as well as behavioral functions of our recorded units.

Spike recovery rates of 84.5% were achieved using the temporal interpolation and spike extraction techniques developed in this study. This high recovery rate allowed us to distinguish behavioral correlates of multiple identified neural units and to differentiate dopamine and neuronal functions. We found that unit activity and dopamine displayed very different patterns of signaling in response to behavioral events in our task. Striatal neurons are known to represent a multitude of parameters related to conflict decision-making (Amemori et al., 2020), learning (Desrochers et al., 2015), and other behavioral functions (Hikosaka et al., 1989; Aosaki et al., 1994, 1995; Apicella, 2002; Yamada et al., 2004; Thorn et al., 2010). On the other hand, dopamine has been largely attributed to a role in reward valuation and prediction error signaling (Schultz et al., 1997). Only recently has dopamine been shown to also provide a prolific representation of motor and sensory variables, outside of simple reward variables (Menegas et al., 2018; Schwerdt et al., 2020; Coddington et al., 2023). A remaining question is how dopamine influences the activity of nearby neurons (Sippy and Tritsch, 2023), such as in the form of plasticity (Brzosko et al., 2019; Shindou et al., 2019), and how these interactions modify or are modified by behavior. The reverse of this, understanding how neuronal activity influences dopamine release (Threlfell et al., 2012), also remains an unresolved question that may be addressed through multimodal measurements such as those demonstrated in this work. Nevertheless, standard trial-averaged computations, as used here, may provide limited insight of such potential interactions. Furthermore, measurements of dopamine and neuronal activity should be performed at local sites given dopamine's spatially heterogeneous operations (Schwerdt et al., 2020; Hamid et al., 2021). Higher recovery rates may be possible for nonsaturating artifacts (i.e., R and RC type) using techniques that may potentially be applied to subtract out the artifact and recover spikes during the artifact (Hashimoto et al., 2002; Wichmann and Devergnas, 2011; Nag et al., 2015; O’Shea and Shenoy, 2018; Banaie Boroujeni et al., 2020).

One of the limitations of our current configuration is that dopamine and neuronal signals are measured from separate, and distal (>1 mm), sensors. Single-sensor systems have been previously developed to combine FSCV transimpedance and EPhys voltage amplifiers with active switching circuitry to measure chemical and voltage signals from the same sensor and site (Takmakov et al., 2011). These have been applied in rodents to successfully measure electrical stimulation evoked dopamine and spike activity (Cheer et al., 2005). Nevertheless, as described in the Introduction, a single-sensor configuration prevents a negative hold potential from being applied in between applied voltage scans, significantly restricting the sensitivity to measure dopamine and other positively charged molecules (Bath et al., 2000). As far as we are aware, measurements of endogenous (i.e., not electrically stimulated) neurochemical signaling with this single-sensor configuration have not been demonstrated. On the other hand, separating the sensors for FSCV and EPhys enabled concurrent recording of naturally occurring dopamine and voltage fluctuations during behavior in rodents (Parent et al., 2017) and monkeys (Schwerdt et al., 2020). This decoupled ECP configuration was therefore used in this work.

Recording dopamine at the same location as the EPhys signal would make it possible to measure the direct interactions of dopamine release on nearby cell firing and vice versa, as discussed above. Dopamine is thought to diffuse only a few microns (Rice et al., 2011), meaning that functional heterogeneity may exist at this fine-grained level. On the other hand, dopamine function in general reward prediction error signaling has been shown to be relatively uniform across a broad millimeter-scale spatial field (e.g., the region traditionally defined as the rodent ventral striatum; Stuber et al., 2008). Recent imaging studies show dopamine release dynamics that are heterogeneous at 40 µm intervals (Hamid et al., 2021). Therefore, dopamine function may vary widely in their spatial scale. On the other hand, these results suggest that even our millimeter separations between FSCV and EPhys recording sensors should provide functionally meaningful associations. Nevertheless, more accurate inferences about interactions may be obtained from the recorded signals the closer the spacing between sampled cross-modalities. Future work is needed to directly characterize the relationship between dopamine and spike signaling as a function of distance between these signals.

So far, two variations of a decoupled ECP system have been reported, as far as we are aware (Parent et al., 2017; Schwerdt et al., 2020). The first system attempted to address or reduce the effects of the FSCV artifacts through several hardware and software modifications. This would allow successful measurements of stimulation and pharmacologically evoked dopamine signals in awake and mobile rodents. A relay circuit was used to isolate FSCV scan voltages from the EPhys recording system. In principle, this would be helpful to prevent large voltages from saturating the EPhys input amplifiers when the electrodes are close to each other. Nevertheless, the relay induced larger artifacts than FSCV scan voltages. A timing signal for the scan voltages was sent to both FSCV and EPhys systems, which allowed readily extracting EPhys recorded signals during the window in between the known scan periods, or interpolating away these periods. An additional window of interpolation (4.5 ms) was added around the scan period, similar to the current work, most likely also to remove the effects of capacitive discharge and amplifier recovery time. Furthermore, a lower scan frequency (5 Hz) was used to produce a wider artifact-free window in which lower-frequency LFPs could be extracted. However, this limits the temporal resolution of the dopamine measurements. Similar to our current work, the second system utilized the standard 10 Hz scan frequency to maintain a higher temporal resolution to capture the fast millisecond dynamics of dopamine release and clearance and did not require any clocked input for transmitting timings of the FSCV scans or other specialized hardware (i.e., relay; Schwerdt et al., 2020). EPhys and FSCV recordings were synchronized through shared behavioral event codes. This system applied a custom-made algorithm to interpolate away these artifacts in the frequency domain, allowing reliable extrapolation of a broad frequency range (0.1 Hz–1 kHz) of LFPs with a high correlation to original waveforms (*R* ∼ 0.99), based on simulated artifacts. However, these algorithms were not capable of reliably extracting extracellular action potentials (i.e., spikes or units) due to the higher-frequency content of these signals (up to ∼8 kHz; Fee et al., 1996). Thus, in this work, time-domain interpolation is used instead of the frequency-domain to preserve the broad frequency content of the spike waveforms, which is imperative to identify individual units via standard spike-sorting methods.

Another potential advantage of our ECP system is the ability to record both unit activity and dopamine signals from the same CF sensor, albeit at different times. This would allow comparisons between spike and dopamine activity from the same site, as measured from different recording sessions. This could be useful for making associations between dopamine and neuronal activity during well-maintained and reproducible behaviors across sessions. Nevertheless, ideally, such measurements would occur at the same time in order to infer temporal correlations between recorded multimodal signals. Measurements from juxtaposed electrode contacts may provide the needed focal site-specific metrics of interacting signals. Standard photolithography and microfabrication processes combined with innovative carbon-based materials (Castagnola et al., 2021; Cuniberto et al., 2021) may make it possible to readily provide juxtaposition of sensors on a micrometer scale. Furthermore, several labs have advanced the ability for FSCV to provide readouts of multiple chemical entities by virtue of each compound's distinct redox voltages (M. D. Nguyen and Venton, 2014; Calhoun et al., 2018; Dunham and Jill Venton, 2020). Finally, advances in materials engineering have shown promise for increasing measurement sensitivity and selectivity for dopamine and other neurochemical compounds (Schmidt et al., 2013; Cuniberto et al., 2021), which may further expand the applications of FSCV and ECP recording systems.

All measurements displayed here were from chronically implanted CF sensors. The FSCV dopamine recording performance of these sensors, independent of EPhys, has been extensively characterized in prior work (Clark et al., 2010; Schwerdt et al., 2018, 2020), where we and others have shown these sensors to provide long-term (more than a year) measurements in rodents (Schwerdt et al., 2018) and monkeys (Schwerdt et al., 2020). Recorded dopamine signals were also shown to be stable from day to day, which may enable future applications to elucidate the role of dopamine in learning and/or in neurodegenerative states in primates. This type of longitudinal recording has already been achieved in rodents, generating breakthroughs in our understanding of how dopamine evolves over extended behavioral processes (Collins et al., 2016; van Elzelingen et al., 2022). Unit recording capabilities of our CF sensors were not extensively characterized in this work, but similar metrics have been generated in a previous report with comparisons to commercial silicon electrode arrays for chronic applications (Patel et al., 2016). There have been tremendous advances in creating high-channel count and high-density multichannel electrode arrays for primates (Trautmann et al., 2023). Future work integrating these arrays with our CF sensors would enable unprecedented ability to record spike activity from a large population of neurons with combined measurements of dopamine release. However, these methods are currently limited to acute settings (i.e., daily penetrations), which may restrict practical combination with FSCV. The combined setup time for preparing and inserting acute EPhys recording arrays alongside FSCV sensors is estimated to take several hours, and successful CF sensor insertion is an inherently low-yield process (i.e., <50% success) due to the fragile nature of the thin 7 µm diameter CF tip.

An important parameter likely to affect the performance of the interpolation algorithm and the interfering effect of coupled artifacts in extracting accurate spike data is the distance between EPhys and FSCV recording sensors. The effects of distance between sensors were not directly tested in our experiments here since a sparse number of electrodes were used and multiple sensors were used for FSCV in our recorded sessions, which would make interpretation of artifact coupling difficult. Future work is needed to quantify the effects of distance by carefully manipulating the distance between recording sensors while independently applying FSCV waveforms to these sensors. It should also be noted that distance is likely not the only influencing property and that other nonlinear properties of the electrode-tissue interface will also affect artifact coupling (Nag et al., 2015).

In summary, we developed a simple system combining standard EPhys and FSCV instrumentation for synchronous measurements of neuronal spike and dopamine signaling for use in nonhuman primates. The system could easily be adopted for use in other species, such as rodents (Parent et al., 2017) and humans (Kishida et al., 2016). Combinatorial methods such as those described here are needed in behavioral experiments to help resolve important questions related to the physiological mechanisms of plasticity and the interactions between neuromodulators and target neurons that regulate ongoing behavior.

## Data Availability

All source data are available at zenodo.org (https://doi.org/10.5281/zenodo.10397754).

## Code Availability

MATLAB code used to analyze data may be found at GitHub as made available through zenodo (https://doi.org/10.5281/zenodo.10955583, https://doi.org/10.5281/zenodo.10955525, https://doi.org/10.5281/zenodo.10397773).

## Synthesis

Reviewing Editor: Nicholas J. Priebe, The University of Texas at Austin Decisions are customarily a result of the Reviewing Editor and the peer reviewers coming together and discussing their recommendations until a consensus is reached. When revisions are invited, a fact-based synthesis statement explaining their decision and outlining what is needed to prepare a revision will be listed below. The following reviewer(s) agreed to reveal their identity: Anita Disney.

Two reviewers evaluated the manuscript "Synchronous Measurements of Extracellular Action Potentials and Neurochemical Activity with Carbon Fiber Electrodes in Nonhuman Primates." Both reviewers agreed that the manuscript describes an important resource for how extracellular spikes and dopamine signals may be collected simultaneously using two separate electrodes. But the reviewers also raised a number of questions with regard to the presentation of the technique as a methods resource. The major questions, detailed below, include adding details of method so that it could be broadly employed and determining the robustness of the technique to experimental differences. These issues are detailed below and should be useful in revising your manuscript for eNeuro or another journal.

Major Issues

1) What is the performance of this method

How well does the system work along the two dimensions of measuring DA signaling and measuring spikes (qualitatively). Does the extracellular recording performance compare with typical tungsten electrodes? How many penetrations were performed vs. how many neurons were successfully isolated? A sense of the tradeoffs here would be useful.

2) How does the present approach differ from a previous paper?

Schwerdt et al 2020 is cited for methods. Were these exact animals and implants were reused (and so the number of CFEs implanted is >15) or is this is the same method but new animals. How many electrodes were employed and how were they spaced? Given the averaging step in the algorithm, were there multiple physiology electrodes, or was there an average across recordings?

3) How robust is the algorithm to varying experimental conditions?

How important is the averaging step in the algorithm? Will this method work with just one electrophysiological and one FSCV electrode? If there are multiple electrodes recording at once, the authors should be able to do this analysis directly by trying out one electrode at a time (no averaging step). It would be helpful to know whether there were electrophysiology electrode positions, relative the FSCV electrode, that did or did not work. These answers would help describe the robustness of the recording method.

4) How do the distances between electrodes impact the recordings?

The authors note that inter-electrode distance is a key variable in determining how artifacts appear in raw traces. They also note that distance between dopamine and spike measurement sites is likely a critical variable in understanding the interaction between chemistry and physiology (because there appear to be dopamine microenvironments in the striatum). Do they have data on recording site spacing to be able to give some more detail on the variations in artifact that arise as a function of electrode spacing? Is there a distance at which the sweep no longer interferes? Is there a distance where the sweep artifact amplitude is small enough to make this algorithm unreliable? How close were the closest electrodes? How does this distance relate to microdomains? Was there a distance that was already too close, despite the sapcing almost certainly being >> microdomain size?

5) Can the authors determine whether FSCV sweeps introduce additional spikes outside the interpolated time windows? One assumes this doesn't happen at >1mm inter-electrode distances, but it seems reasonable to check and the means to at least try to do so should be available (e.g. by recording with/without FSCV in the same session).

6) As a technical resource it is important to articulate why this approach is has an advantage over alternatives. One approach might be to take the existing CF probe (with no spiking measurements) and then insert a separate electrode / linear electrode array nearby. Could a Plexon V-probe or even a Neuropixels probe in an adjacent grid hole 1 mm away, and get more neurons? Is this possible given the two sensors on the CF probe are already >1mm apart? Alternatively, why not collect the DA signal and the electrophysiological signal on two different days? The manuscript would be improved by considering these alternative methods. Does the DA signaling vary from day to day? Does the system work reliably enough trials to look at within-session learning? How critical is it that the DA signal be measured in the same location as the electrophysiological signal?

7) The method sections is written very linearly, but the rationale is left somewhat implicit. It would benefit from a bit of guidance that walks the reader through the point of each operation. For example, much of the section "Temporal interpolation methods to extract spike data" is dedicated to finding which periods of time in the recording to blank out (or interpolate a line over). The methods are fine / reasonable, but there is a lot of detail in which one could try to recover spikes during these windows. In the end, however, the waveform is simply replaced with a flat line that will flatten in the high-pass filtering. This section could be much improved with a explicit preface that explains the approach at a high level, e.g., find the artifact peaks, remove them, then run a typical spike extraction procedure.

Minor Issues

1) The time scales in figure 1 should be referenced to something in the trial, e.g., make time 0 the cue. Having the time start at a meaningless offset (18.7 seconds? from what?) isn't useful to the reader. Having the ticks in integer milliseconds would also make it easier to see the durations.

2) The spike raster in 6E is very difficult to see the modulation. Consider having separate (black) rasters for the two reward conditions to make the structure easier to see. It's a mess of blurry red and blue dots. Same with 6B, though there the right response is easier to pick out.
