# Supplementary figures and images for: Synchronous Measurements of Extracellular Action Potentials and Neurochemical Activity with Carbon Fiber Electrodes in Nonhuman Primates
Source: eNeuro. 2024 Jul 4;11(7):ENEURO.0001-24.2024. doi: 10.1523/ENEURO.0001-24.2024 (PMC11232371; doi:10.1523/ENEURO.0001-24.2024)

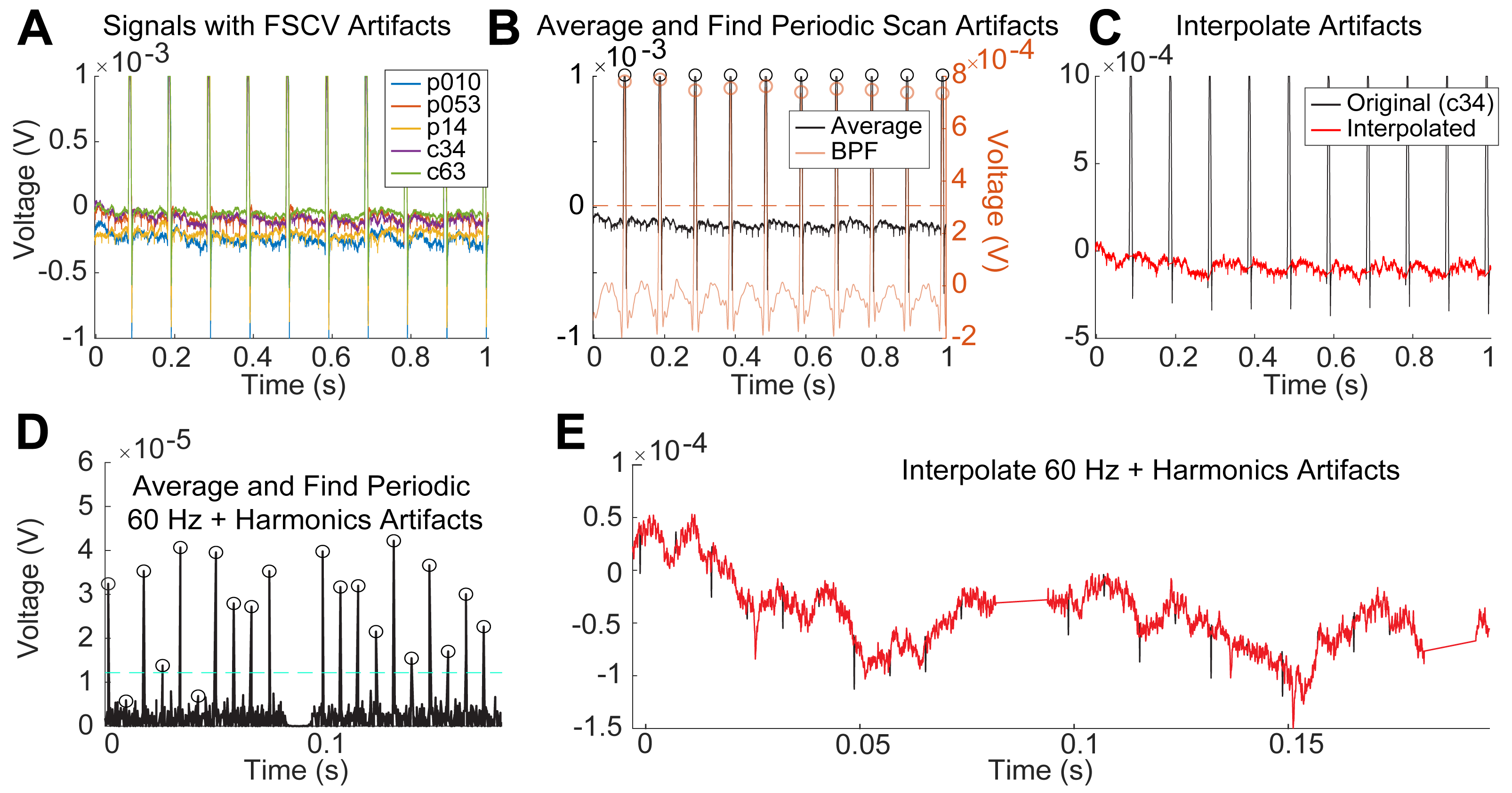

Supplement: Figure 3-1 — Same as Fig. 3 but demonstrating an example with 60 Hz + harmonics noise and the additional steps to remove these signals. (D) After high pass filtering, the noise was enhanced. (E) Unit spike activity is more clearly discernible after interpolating both 60 Hz harmonics noise and the FSCV artifacts. Download Figure 3-1, TIF file. [file eneuro-11-ENEURO.0001-24.2024-s002.tif]
